# You can have it all: How the interplay between SnRK1 and RBOH1 promotes nitrate uptake in tomato

**DOI:** 10.1093/plcell/koae325

**Published:** 2024-12-13

**Authors:** Margot Raffeiner

**Affiliations:** Assistant Features Editor, The Plant Cell, American Society of Plant Biologists; Faculty of Biology and Biotechnology, Ruhr University Bochum, Bochum 44801, Germany

In the modern world, everything seems to be geared toward optimization. “Higher, faster, further” has become the dominant mindset, although you should ask yourself from time to time whether this is really the right way to go. Nevertheless, for some organisms, optimization is a must. Plants, for example, need to optimize the uptake of essential nutrients from the soil to ensure their healthy growth, adaptation to various stresses, and their reproductive success.

Nitrogen (N) is one such essential plant macronutrient. It is absorbed in 2 major forms, nitrate (NO_3_^−^) and ammonium (NH_4_^+^), both of them being present in limited amounts in agricultural and natural ecosystems (Ye et al. 2022). To optimize N absorption under fluctuating conditions, plants have developed high- and low-affinity transport systems (HATS and LATS, respectively). The LATS Nitrate Transporter 1 (NRT1) and HATS Nitrate Transporter 2 (NRT2) protein families are responsible for efficient nitrate absorption and transport, while the HATS AMT transporter superfamily mediates ammonium assimilation (Ye et al. 2022).

A shortage in N availability in the soil can cause severe nutritional stress to a plant. It has been proposed that Arabidopsis employs at least 2 master regulators of diverse plant stress responses: the Sucrose-nonfermenting 1-related kinase 1 (SnRK1) and the NADPH oxidase/respiratory burst oxidase homolog (RBOH) proteins (Chen et al. 2017; Safi et al. 2021; Mittler et al. 2022). RBOH proteins produce reactive oxygen species (ROS), but little was known about the functional relationship between ROS production and N assimilation. While it has been shown that SnRK1 regulates N starvation responses via the autophagy pathway, it is unclear whether this kinase might influence other N uptake pathways to boost plant tolerance toward low N stress (Chen et al. 2017).

In recent work on the important crop plant tomato (*Solanum lycopersicum* L. cv. Ailsa Craig), **Xuelian Zheng and colleagues (Zheng et al. 2024)** addressed those questions to provide meaningful insights into the regulatory mechanisms that govern plant low N stress tolerance.

By subjecting tomato knockout mutants for *SnRK1α1*, the most functionally active SnRK1 subunit, to low N treatment, the authors observed specific phenotypes linked to this stress. These plants displayed a more pronounced leaf yellowing, reduced chlorophyll content and biomass, as well as decreased N and carbon contents compared with wild-type plants or *SnRK1α1* overexpressing lines. In addition, *snrk1α1* mutants failed to efficiently induce N transporter gene expression and to accumulate high amounts of ROS under N starvation conditions, indicating that both stress-regulating measures are functionally dependent on SnRK1α1 kinase activity.

To investigate this relationship in more detail, Zheng and colleagues analyzed the activity of tomato RBOH1, the NADPH oxidase responsible for the production of the ROS hydrogen peroxide (H_2_O_2_), which was found to be suppressed in *snrk1α1* mutants. Using a virus-induced gene silencing approach to dampen *RBOH1* gene expression in tomato wild-type and *SnRK1α1* overexpressing plants followed by quantitative assessment of H_2_O_2_ content in tomato roots, the authors confirmed that elevated ROS production linked to SnRK1α1 activity was dependent on RBOH1, which most likely acts downstream of the SnRK1 kinase. After validating a physical interaction between SnRK1α1 and RBOH1, the authors proceeded with a detailed mechanistic analysis of this interplay under low N conditions. This revealed that SnRK1α1 phosphorylates and thereby activates RBOH1 to enhance H_2_O_2_ production. H_2_O_2_ subsequently induces oxidative post-translational modifications on the important transcription factor in N response regulation TGA4, which enhances its binding to *NRT* gene promoters and promotes *NRT* gene expression to ultimately improve nitrate uptake (see Figure).

**Figure.**
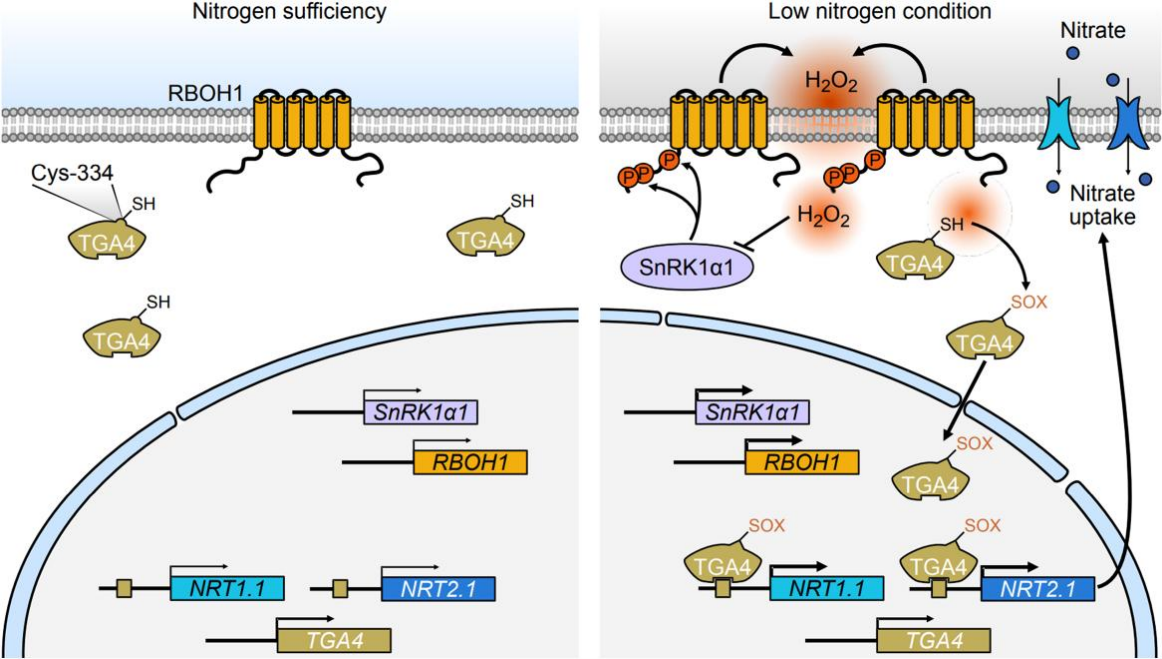
The SnRK1α1-RBOH1 module induces H_2_O_2_ production to promote low N tolerance in tomato. This model illustrates how, under low N conditions, the SnRK1α1 kinase phosphorylates the NADPH oxidase RBOH1 at specific residues, thereby inducing the production of the reactive oxygen species H_2_O_2_s. H_2_O_2_ subsequently oxidizes the transcription factor TGA4 via oxidative posttranslational modifications (oxi-PTMs). Oxidized TGA4 binds to promoters and boosts expression of the nitrate transporter genes *NRT1.1* and *NRT2.1*, which ultimately leads to optimized nitrate absorption. To balance the redox status in the cell, it is proposed that excessive amounts of H_2_O_2_ are sensed and serve to inhibit SnRK1α1 activity (indicated by the inhibitory arrow on the right side of the figure). Reproduced from Zheng et al. (2024), Figure 9.

Taken together, this study provides a detailed molecular mechanism that describes how plants can optimize nitrate absorption to cope with N starvation-induced nutritional stress.

## Data Availability

Data is available at https://doi.org/10.1093/plcell/koae321.

## Dive Curated Terms

The following phenotypic, genotypic, and functional terms are of significance to the work described in this paper:

TGA4 Gramene: Solyc04g054320
TGA4 Araport: Solyc04g054320

NADPH CHEBI: CHEBI:16474

nitrogen CHEBI: CHEBI:25555

RBOH1 Gramene: Solyc08g081690
RBOH1 Araport: Solyc08g081690
